# P-glycoprotein confers acquired resistance to 17-DMAG in lung cancers with an ALK rearrangement

**DOI:** 10.1186/s12885-015-1543-z

**Published:** 2015-07-29

**Authors:** Hee Joung Kim, Kye Young Lee, Young Whan Kim, Yun Jung Choi, Jung-Eun Lee, Chang Min Choi, In-Jeoung Baek, Jin Kyung Rho, Jae Cheol Lee

**Affiliations:** 1 Department of Internal Medicine, Konkuk University Medical Center, Seoul, South Korea; 2 Department of Internal Medicine, Division of Pulmonary and Critical Care Medicine, Seoul National University College of Medicine, Seoul, South Korea; 3 Department of Pulmonary and Critical Care Medicine, Asan Medical Center, College of Medicine, University of Ulsan, Seoul, South Korea; 4 Department of Oncology, Asan Medical Center, College of Medicine, University of Ulsan, 86 Asanbyeongwon-gil, Songpa-gu, Seoul 138-736 South Korea; 5 Asan Institute for Life Sciences, Asan Medical Center, College of Medicine, University of Ulsan, Seoul, South Korea

**Keywords:** Lung cancer, ALK, Heat shock protein 90, Resistance, P-glycoprotein

## Abstract

**Background:**

Because anaplastic lymphoma kinase (ALK) is dependent on Hsp90 for protein stability, Hsp90 inhibitors are effective in controlling growth of lung cancer cells with ALK rearrangement. We investigated the mechanism of acquired resistance to 17-(Dimethylaminoethylamino)-17-demethoxygeldanamycin (17-DMAG), a geldanamycin analogue Hsp90 inhibitor, in H3122 and H2228 non-small cell lung cancer cell lines with ALK rearrangement.

**Methods:**

Resistant cell lines (H3122/DR-1, H3122/DR-2 and H2228/DR) were established by repeated exposure to increasing concentrations of 17-DMAG. Mechanisms for resistance by either NAD(P)H/quinone oxidoreductase 1 (NQO1), previously known as a factor related to 17-DMAG resistance, or P-glycoprotein (P-gp; ABCB1/MDR1) were queried using RT-PCR, western blot analysis, chemical inhibitors, the MTT cell proliferation/survival assay, and cellular efflux of rhodamine 123.

**Results:**

The resistant cells showed no cross-resistance to AUY922 or ALK inhibitors, suggesting that ALK dependency persists in cells with acquired resistance to 17-DMAG. Although expression of NQO1 was decreased in H3122/DR-1 and H3122/DR-2, NQO1 inhibition by dicumarol did not affect the response of parental cells (H2228 and H3122) to 17-DMAG. Interestingly, all resistant cells showed the induction of P-gp at the protein and RNA levels, which was associated with an increased efflux of the P-gp substrate rhodamine 123 (Rho123). Transfection with siRNA directed against *P-gp* or treatment with verapamil, an inhibitor of P-gp, restored the sensitivity to the drug in all cells with acquired resistance to 17-DMAG. Furthermore, we also observed that the growth-inhibitory effect of 17-DMAG was decreased in A549/PR and H460/PR cells generated to over-express P-gp by long-term exposure to paclitaxel, and these cells recovered their sensitivity to 17-DMAG through the inhibition of P-gp.

**Conclusion:**

P-gp over-expression is a possible mechanism of acquired resistance to 17-DMAG in cells with ALK rearrangement.

**Electronic supplementary material:**

The online version of this article (doi:10.1186/s12885-015-1543-z) contains supplementary material, which is available to authorized users.

## Background

Targeted therapy using tyrosine kinase inhibitors against oncogenic driver mutations in non-small cell lung cancer (NSCLC) has been developed to enhance selective cytotoxicity against tumor cells. The echinoderm microtubule-associated protein-like 4 - anaplastic lymphoma kinase gene (EML4-ALK) fusion oncoprotein, which arises from an inversion within chromosome 2p and results in constitutive kinase activity by dimerization of ALK, represents a major molecular target in lung cancer [1]. Although, ALK-rearranged lung cancer accounts for only 3-7 % of NSCLC since its discovery in 2007, this population could represent more than 70,000 new cases worldwide annually. Furthermore, the detection rates are higher in the selected subgroup for genetic screening based on clinical features commonly associated with ALK-rearrangement, including never or light smoking history, adenocarcinoma histology, and wild-type epidermal growth factor receptor (EGFR) and KRAS status [2].

Crizotinib is an oral-administered multitargeted small molecule tyrosine kinase inhibitor, which inhibits mesenchymal epithelial transition growth factor (c-MET) as well as ALK phosphorylation that is recommended as a first-line treatment option for patients with locally advanced or metastatic NSCLC who have the ALK gene rearrangement [3]. Crizotinib shows superiority over standard chemotherapy in progression-free survival (7.7 vs. 3.0 mo) and objective response rate (65 % vs. 20 %) in patients with previously treated, advanced NSCLC with ALK rearrangement [4]. However, despite the successful initial response, most patients inevitably encounter the development of acquired resistance while being treated with crizotinib [5, 6] similar to EGFR tyrosine kinase inhibitors (TKIs). There are multiple resistance mechanisms such as various acquired mutations, which hamper drug binding, oncogenic bypass through EGFR or c-KIT activation [5, 7], and induction of the epithelial-mesenchymal transition [8]. Ceritinib, a second-generation ALK inhibitor, is effective in patients resistant to crizotinib as well as crizotinib-naive patients and is approved by the US Food and Drug Administration for patients who have tumor progression or are intolerant of crizotinib [9]. The other second-generation ALK inhibitors such as CH5424802, AP26113, ASP3026, X-396, and TSR-011 are undergoing phase I or II clinical trials [10]. In addition, heat shock protein (HSP) 90 inhibitors are suggested as therapeutic options to overcome resistance on the basis of anti-tumor activity in preclinical models of ALK-driven lung cancer [11, 12] and small-scale clinical trials on ALK-positive lung cancers [13].

Hsp90 is a molecular chaperone that plays an important role in the modification and stabilization of a variety of proteins implicated in tumor cell proliferation and survival. Both EGFR and EML4–ALK fusions, which are known to be major oncogenic drivers in NSCLC, are client proteins for Hsp90 [14, 15, 11]. Therefore, Hsp90 could be an alternative therapeutic target instead of direct kinase inhibition in ALK-driven lung cancer. *In vivo* and *in vitro* studies demonstrated that treatment with Hsp90 inhibitors such as 17-DMAG, ganetespib (STA-9090), or IPI-504 reduced protein levels of the ALK fusion protein, enhanced cell death, led to tumor regression, and prolonged survival of xenograft models [14, 15, 12]. Antitumor activity also has been observed in phase I and II clinical trials with ganetespib or IPI-504 [16, 13], and a number of Hsp90 inhibitors - both as monotherapies and in combination with ALK tyrosine kinase inhibitors - are undergoing clinical trials for ALK-positive lung cancer patients.

Although many studies have identified resistance factors associated with ALK inhibitors, the mechanisms of resistance to Hsp90 inhibitors are poorly understood. Clarification of the resistance mechanisms relevant to ALK-positive lung cancer may be important to find ways to overcome drug resistance. In this study, we generated resistant cells by treating ALK-positive cells with increasing concentrations of 17-DMAG, and investigated the mechanism of their resistance.

## Methods

### Cell culture and reagents

The human NSCLC cell line H2228, A549 and H460 were purchased from the American Type Culture Collection (Rockville, MD). The H3122 cell line was a gift from Adi F. Gazdar (UT Southwestern, Dallas, TX). Cells were cultured in 10 % fetal bovine serum (FBS) supplemented with 100 U/mL penicillin and 100 μg/mL streptomycin (Invitrogen, Carlsbad, CA) at 37 °C in an atmosphere with 5 % CO_2_. Crizotinib, TAE-684, 17-DMAG, AUY-922, and verapamil hydrochloride were obtained from Selleck Chemicals Co. Ltd (Houston, TX). The 3-(4,5-dimethylthiazol-2-yl)-2,5-diphenyltetrazolium bromide (MTT) solution, 3,3’-methylene-bis(4-hydorxycoumarin) (dicumarol), and Rho123 were purchased from Sigma-Aldrich (St. Louis, MO).

### Establishment of 17-DMAG or paclitaxel resistance in NSCLC cells

Cells resistant to 17-DMAG or paclitaxel were developed by chronic, repeated exposure to each drug. Over a period of 6 months, cells were continuously exposed to increasing concentrations of the drug in culture and the surviving cells were cloned. These cells could survive exposure >50 nM of 17-DMAG or >100 nM of paclitaxel. In all studies, resistant cells were cultured in drug-free medium for >1 week to eliminate the effects of 17-DMAG or paclitaxel.

### MTT assay

Cells were seeded onto 96-well plates and incubated overnight, and then treated with their respective agents for an additional 3 days. Cell viability was determined using the previously described MTT-based method [17]. Each assay consisted of eight replicate wells and was repeated at least three times. Data were expressed as the percent survival of the control, which was calculated using absorbance after correcting for background noise.

### Western blot analysis

Whole cell lysates were prepared using EBC lysis buffer (50 mM Tris–HCl [pH 8.0], 120 mM NaCl, 1 % Triton X-100, 1 mM EDTA, 1 mM EGTA, 0.3 mM phenylmethylsulfonyl fluoride, 0.2 mM sodium orthovanadate, 0.5 % NP-40, and 5 U/mL aprotinin) and centrifuged. Proteins were separated using SDS-PAGE and transferred to PVDF membranes (Invitrogen) for western blot analysis. Membranes were probed with antibodies against p-ALK (Tyr1604), ALK, p-Akt (Ser473), P-gp (all from Cell Signaling Technology, Beverly, MA), Akt, p-Erk (Thr202/Tyr204), Erk, HSF1, Hsp90, Hsp70, Hsp27, NQO1, and β-actin (all from Santa Cruz Biotechnology, Santa Cruz, CA) as the first antibody, and then membranes were treated with horseradish peroxidase-conjugated secondary antibody. All membranes were developed using an enhanced chemiluminescence system (Thermo Scientific, Rockford, IL).

### Detection of *NQO1* polymorphism

DNA purification and detection of the gene polymorphism were performed according to the previously reported methods [18]. Briefly, for the amplification of the *NQO1* gene fragment (230 bp), a pair of forward and reverse primers were as follows; 5’-TCCTCAGAGTGGCATTCTGC-3’ and 5’-TCTCCTCATCCTGTACCTCT-3’. The amplification was carried out by using AccuPower TagPCR PreMix (Bioneer Corp., Daejeon, Korea). Each PCR mixture contained forward and reverse primers (each 0.5 pmoL) and 50 ng of genomic DNA in a final volume of 20 μL. PCR conditions consisted of initial denaturing at 94 °C for 5 min, 35 amplification cycles (95 °C for 30 s, 58 °C for 30 s, and 72 °C for 30 s), and a final extension at 72 °C for 5 min. For restriction fragment length polymorphism (RFLP), the amplified fragments were digested with Hinf1 (Thermo Scientific) and analyzed on agarose gel electrophoresis. The wild-type (Pro187Ser) allele of *NQO1* was identified by a 191 bp band while the homozygous variant (Ser/Ser) and the heterozygous variant (Pro/Ser) displayed only a 151 bp band and two bands (191 bp and 151 bp), respectively.

### Quantitative reverse transcription-polymerase chain reaction (RT-PCR)

Total RNA isolation and cDNA synthesis were performed using the RNA mini-kit protocol (Qiagen Inc., Valencia, CA) and Accupower RT mix reagent, according to the manufacturer’s instructions (Bioneer Corp., Daejeon, Korea). The oligonucleotide sequences for amplification were as follows: forward primer 5’-AAGCAGTGCTTTCCATCA-3’ and reverse primer 5’-TCCTGCCTGGAAGTTTAG-3’ for *NQO1*; forward primer 5’-AGGCCTATTACCCCAGCAT-3’ and reverse primer 5’-CGATCTTGGCGATGTTGATG-3’ for *MRP1*; forward primer 5’-AATAGCACCGACTATCCA-3’ and reverse primer 5’-GTGGGATAACCCAAGTTG-3’ for *MRP2*; forward primer 5’-TGAGATCATCAGTGATACTAA-3’ and reverse primer 5’-ATGCGGCTCTTGCGGAG-3’ for *MRP3*; forward primer 5’-GTACATTAACATGATCTGGTC-3’ and reverse primer 5’-CGTTCATCAGCTTGATCCGAT-3’ for *MDR1*; forward primer 5’-GCGAGAAGATGACCCAGATC-3’ and reverse primer 5’-CCAGTGGTACGGCCAGAGG-3’ for *β-actin*; forward primer 5’-GAGTCAACGGATTTGGTCGT-3’ and reverse primer 5’-TTGATTTTGGAGGGATCTCG-3’ for glyceraldehydes-3-phosphate dehydrogenase (*GAPDH*). PCR cycling conditions were as follows: 94 °C for 60 s and primer annealing for 60 s, elongation at 72 °C, for a total of 30–35 cycles (*NQO1*, *MRP1*, *MRP2*, *MRP3*, *MDR1*) and 25 cycles (*β-actin*, *GAPDH*), respectively. A final extension was terminated by a final incubation at 72 °C for 10 min. Annealing temperatures were 49 °C for *MRP2*, 55 °C for *β-actin* and *MDR1*, 58 °C for *MRP1* and *MRP3*, and 60 °C for *NQO1* and *GAPDH*.

### Rhodamine 123 efflux assay

Cells were incubated with or without 1 μM Rho123 for 1 h. The cells were then washed twice in ice-cold medium and harvested (Rho123 accumulation of cells) or incubated for 3 h in Rho123-free medium. All samples were kept at 4 °C until cytometric analysis was performed. Fluorescence of Rho123 was analyzed on a FACScalibur flow cytometer and processed by Cell Quest Software (BD Bioscience, San Jose, CA). Rho123 efflux was measured by counting cells in the M1 region of the plot and calculated as the percentage of cells in the M1 region of the plot.

### Transfection of small interfering RNA

Small interfering RNA (siRNA) oligonucleotides specific to *P-gp* and the siRNA control were purchased from Santa Cruz Biotechnology. Introduction of siRNA was performed using Lipofectamine 2000 (Invitrogen) in accordance with the manufacturer’s instructions. After transfection, the suppression of P-gp was determined by western blot analysis. For the MTT assay, cells were seeded onto 96-well plates after siRNA transfection, and then treated with the indicated drugs for 72 h.

## Results

### Cells with acquired resistance to 17-DMAG are sensitive to ALK inhibitors

**Fig. 1:**
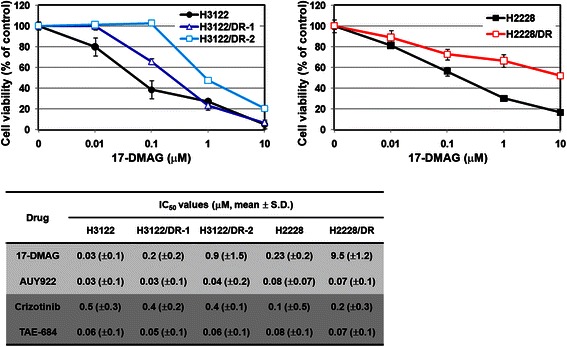
Establishment of acquired resistance to 17-DMAG in H3122 and H2228 cells. Cell viability and the drug concentrations responsible for 50 % growth inhibition were determined using the MTT assay. Cells were treated with 17-DMAG, AUY922, crizotinib, or TAE-684 for 72 h. The values were calculated with data from at least three independent experiments. Bars represent standard deviation. Resistant cells to 17-DMAG were still sensitive to AUY922 and ALK tyrosine kinase inhibitors such as crizotinib and TAE-684

Several 17-DMAG-resistant sublines, derived from parental H3122 and H2228 cell lines, were established by stepwise selection using increasing concentrations of 17-DMAG, as described in the Materials and Methods section. The sublines with acquired resistance included two clones (H3122/DR-1 and H3122/DR-2) from H3122 and only one clone (H2228/DR) from H2228 because H2228 cells did not form colonies. As shown in Fig. 1, all resistant cells showed approximately 10-fold higher resistance to 17-DMAG than the parental cells, although H3122/DR cells acquired a higher resistance than H2228/DR cells. Interestingly, 17-DMAG-resistant cells showed no cross-resistance to AUY922, a potent, novel synthetic resorcinylic isoxazole amide inhibitor of Hsp90, as well as to ALK inhibitors. These results demonstrate that 17-DMAG-resistant cells maintain their ALK dependency.

**Fig. 2:**
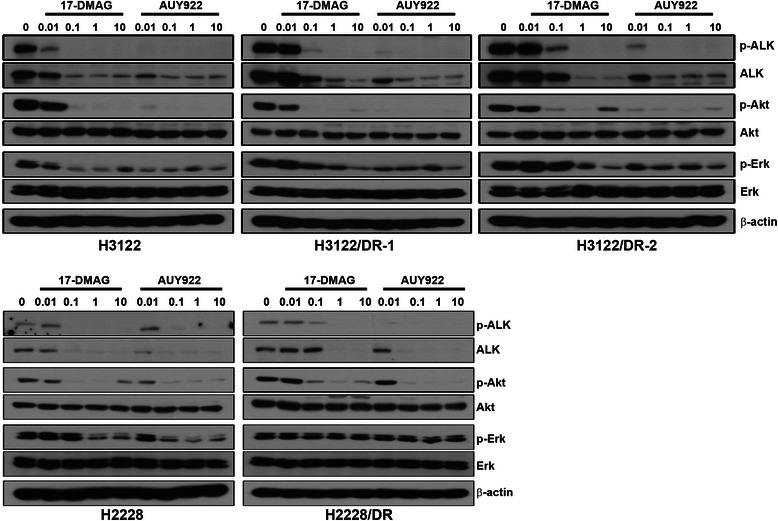
Modulation of ALK signaling in parental and 17-DMAG-resistant cells. Cells were treated with the indicated concentrations of 17-DMAG or AUY922 for 6 h. The molecules of ALK-related signaling activity were detected by western blot analysis

The EML4-ALK fusion oncoprotein has been established as a client protein of Hsp90, and Hsp90 inhibitors were shown to lower ALK levels in cells in culture and xenografts, leading to growth inhibition [15, 12]. To evaluate why the resistant cells were sensitive to AUY922, we examined the modulation of ALK signaling using western blot analysis. Following AUY922 treatment we observed that the suppression of ALK activity, Akt, and Erk was similar in both parental and 17-DMAG-resistant cells (Fig. 2). Unlike that observed in parental cells, the activity of ALK in all 17-DMAG-resistant cells was maintained in the presence of 0.1 μM 17-DMAG, showing that the inhibitory effect of 17-DMAG on ALK signaling was lower in all resistant cells than in parental cells. These findings may not result from the reduction of drug binding affinity because known drug-resistance mutations within the N-terminal domain of Hsp90 containing its ATP-binding site were not detected (data not shown). Taken together, these results indicate that the acquisition of 17-DMAG resistance may be caused by failing to completely abolish ALK activity.

### NQO1 expression is not associated with acquired resistance to 17-DMAG

**Fig. 3:**
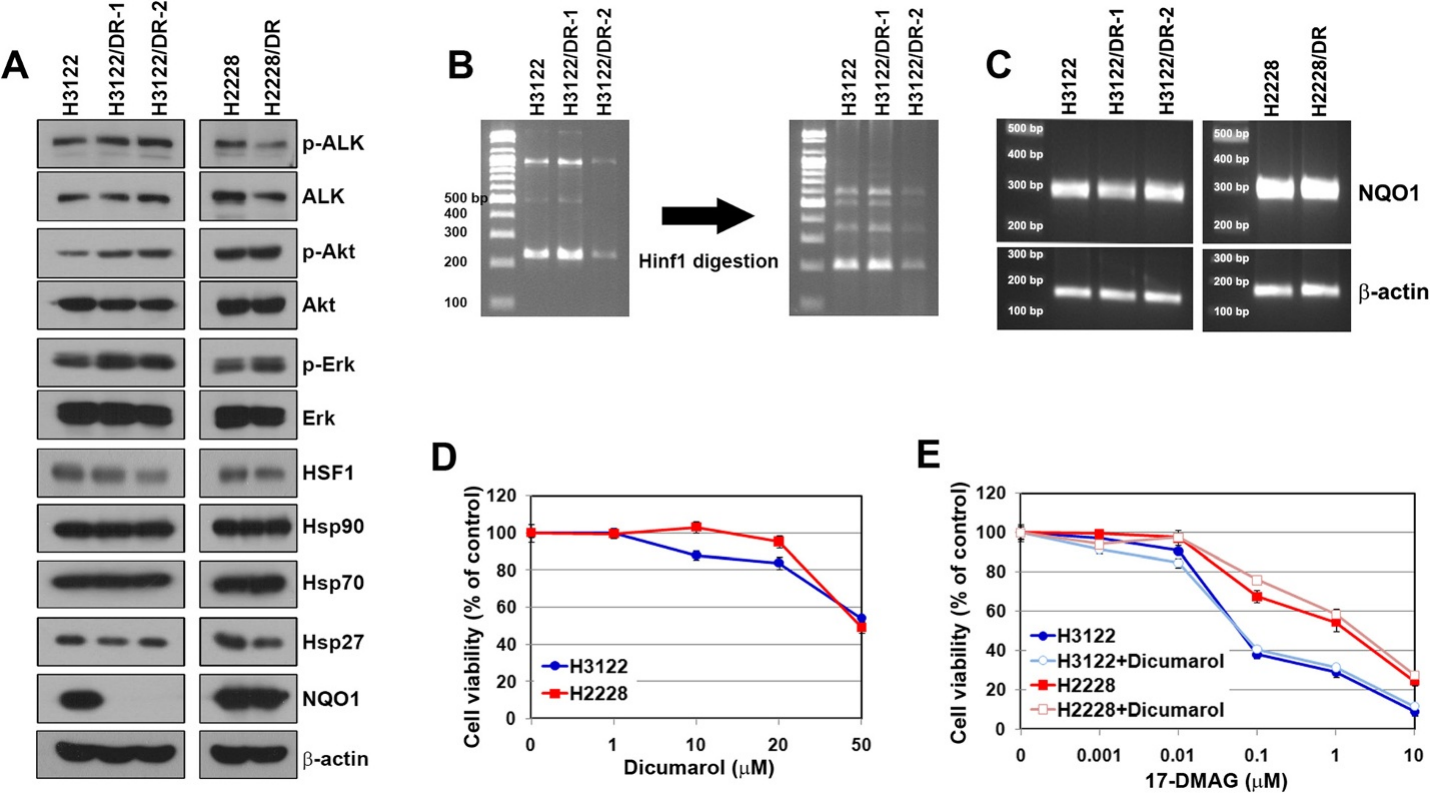
Assessment of NQO1 expression in the parental and acquired resistant cells. **a** Lysates from each cell line were subjected to western blot analysis. The indicated antibodies were used to evaluate the level of proteins involved in resistance to Hsp90 inhibitors. **b** *NQO1* gene fragments were amplified (left panel) and digested by Hinf1 endonuclease (right panel) to assess for gene variations. **c** Total mRNA levels of *NQO1* were measured by quantitative RT-PCR. Cells were treated with the indicated concentrations of dicumarol (**d**) or a combination of 17-DMAG with 20 μM dicumarol (**e**). Cell viability was determined using the MTT assay

NQO1 expression, induction of other heat shock proteins (Hsp70 and Hsp27), or activation of heat shock factor 1 (HSF1) have led to resistance to Hsp90 inhibitors [19–22]. We first examined these factors at the basal protein level. No difference was detected in the above-mentioned factors between parental and resistant cells, but NQO1 expression was significantly decreased in H3122/DR-1 and H3122/DR-2 cells, although its levels were unaltered in H2228/DR cells (Fig. 3a). NQO1 protein levels are influenced by a single nucleotide polymorphism (C609T) in the *NQO1* gene [21]. However, we did not detect any polymorphism in the *NQO1* gene in H3122/DR-1 and H3122/DR-2 cells (Fig. 3b). In addition, both parental and resistant cells showed a similar level of *NQO1* mRNA expression (Fig. 3c). To further investigate the relationship between NQO1 and sensitivity to 17-DMAG, we treated cells with dicumarol, a selective inhibitor of NQO1. Parental cells did not display cytotoxicity to dicumarol until the concentration reached 20 μM (Fig. 3d), and thus cells were treated with 17-DMAG after dicumarol treatment. All cells showed an identical sensitivity to 17-DMAG regardless of dicumarol treatment (Fig. 3e). These results indicate that reduction of NQO1 expression or activity is unlikely to be the main resistance mechanism in ALK-positive cells.

### The induction of P-gp leads to 17-DMAG resistance

**Fig. 4:**
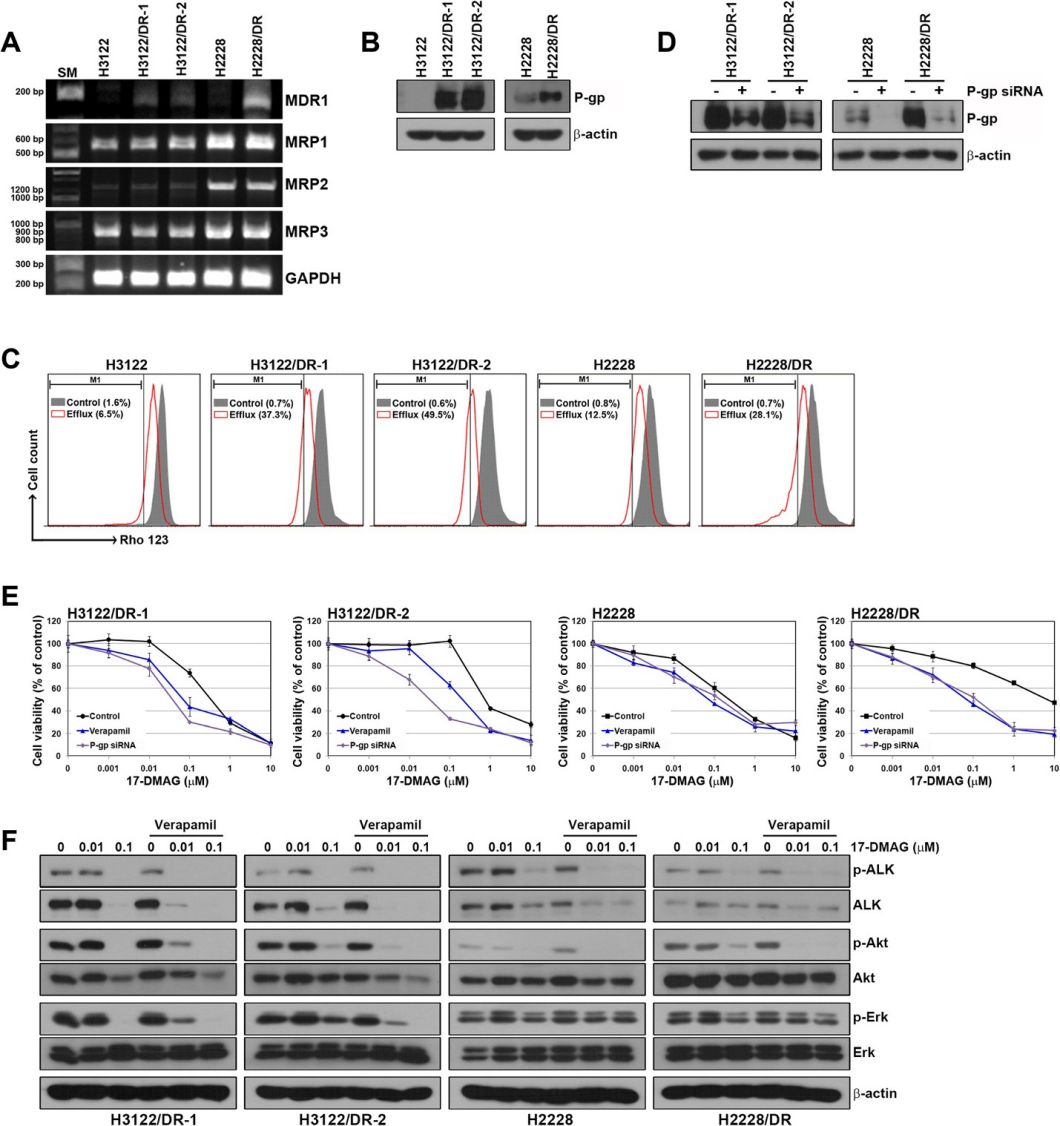
Induction of P-gp/MDR1 expression in 17-DMAG-resistant cells. **a** Detection of *MRPs* and *MDR1* mRNA was performed by quantitative RT-PCR. The sizes of the PCR products were 525 bp (*MRP1*), 1254 bp (*MRP2*), 828 bp (*MRP3*), 157 bp (*MDR1*), and 230 bp (*GAPDH*). **b** P-gp expression was assessed using western blot analysis. **c** Cells were treated with 1 μM Rho123 for 1 h (gray peak) and then incubated with Rho123-free media for 3 h (red blank peak). Rho123 fluorescence was analyzed by flow cytometry. Rho123 efflux was measured by counting cells at the left of the dashed line of the plot (M1 region). **d** Control and *P-gp* siRNAs (100 nM) were introduced into parental or resistant cells, and P-gp silencing was confirmed by western blot analysis. **e** Cells were treated with the indicated concentrations of 17-DMAG after transfection of *P-gp* siRNA or pretreatment with 5 μM verapamil. Cell viability was measured 72 h later using the MTT assay. **f** Cells were pretreated with or without 5 μM verapamil and then treated with the indicated concentrations of 17-DMAG for 6 h. The proteins involved in ALK-related signaling were detected by western blot analysis

Previous studies have shown that the geldanamycin (17-AAG) and ansamycin derivatives of Hsp90 inhibitors are inactive in P-gp/MDR1- and/or MRP1-expressing cell lines [23–25]. We examined the mRNA and protein level of the major transporters involved in drug pumping. As shown in Fig. 4a and b, mRNA levels of multidrug resistance proteins (*MRP1-3*) showed no difference between parental and resistant cells, but those of P-gp were significantly increased in all resistant cells. We next used Rho123 as a surrogate indicator to determine the activity of P-gp. P-gp-mediated transport, indicated by intracellular decrease in Rho123 fluorescence, was studied using flow cytometry. Compared with parental cells (H3122 and H2228), a significant decrease in intracellular Rho123 was observed in all resistant cells, and the mean percentage of Rho123 efflux (M1 region) from cells was 6.5 % (H3122), 37.3 % (H3122/DR-1), 49.5 % (H3122/DR-2), 12.5 % (H2228) and 28.1 % (H2228/DR) (Fig. 4c). To determine whether the induction of P-gp affected the sensitivity to 17-DMAG, we used an siRNA and verapamil, a selective inhibitor of P-gp [26, 27], to inhibit the expression and activity of P-gp, respectively. siRNA treatment effectively suppressed the P-gp expression (Fig. 4d), and there were no significant changes in the rate of proliferation after treatment with 5 μM verapamil in all cell lines (data not shown). The inhibition of P-gp (by suppression of protein expression or reduction of activity) restored responsiveness to 17-DMAG in all resistant cells (Fig. 4e). Interestingly, H2228 cells showed a slightly increased sensitivity to 17-DMAG through the inhibition of P-gp. When resistant cells were pretreated with verapamil, sensitivity to 17-DMAG was restored, and the inhibition of ALK signaling by 17-DMAG was equal to that of the parental cells (Fig. 4f).

**Fig. 5:**
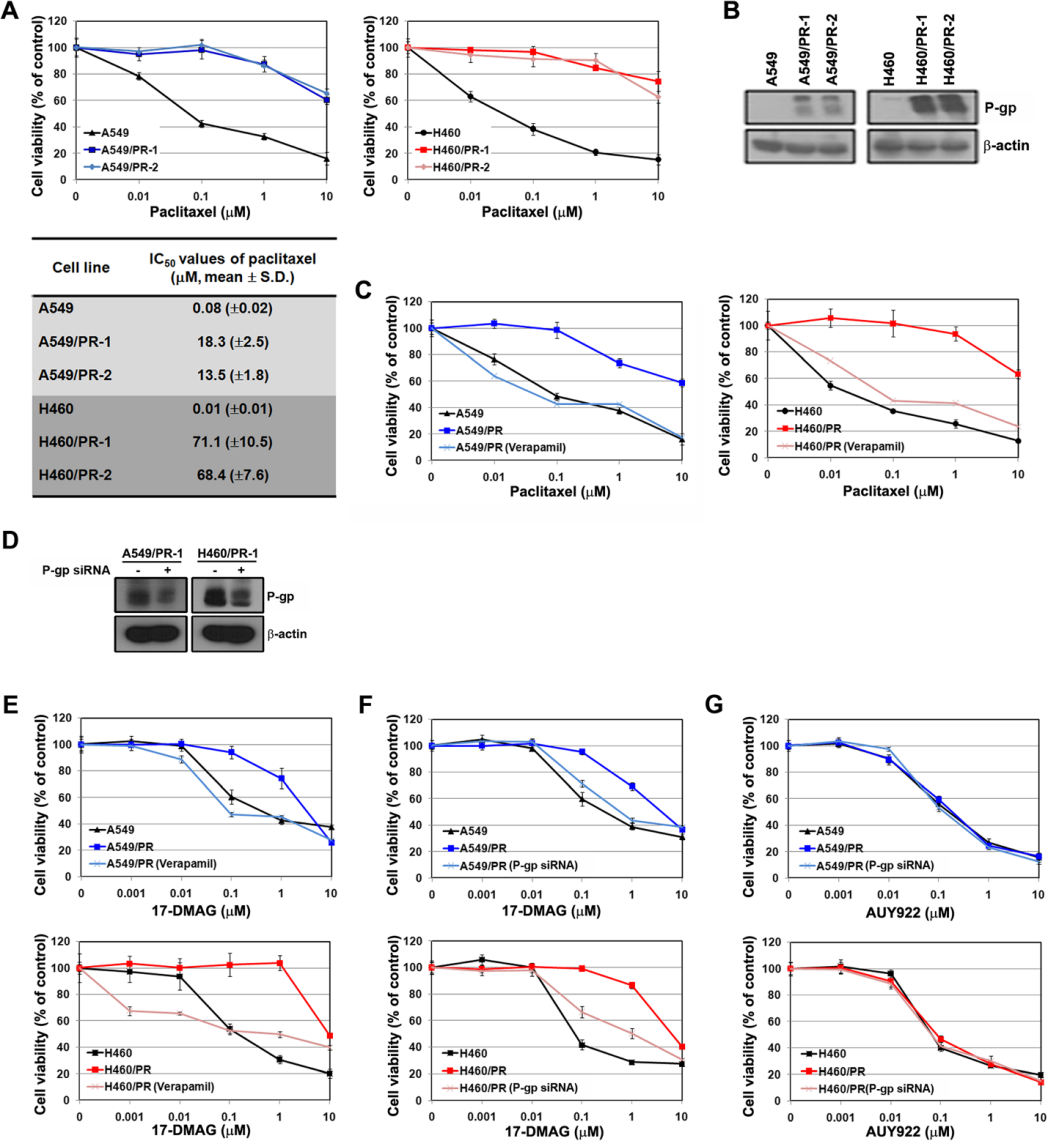
Induction of P-gp/MDR1 expression in paclitaxel-resistant cells. **a** The response to paclitaxel was assessed as described in Fig. 1. **b** P-gp expression was analyzed using western blot analysis. **c** Cells were pretreated with or without 5 μM verapamil and then treated with the indicated concentrations of paclitaxel. After 72 h, cell viability was measured using the MTT assay. **d** Control or *P-gp* siRNAs (100 nM) were introduced into resistant cells, and P-gp silencing was confirmed by western blot analysis. **e, f** and **g** Cells were treated with the indicated concentrations of 17-DMAG or AUY922 after transfection of P-gp siRNA or pretreatment with 5 μM verapamil. Cell viability was measured 72 h later using the MTT assay

We also found that the induction of P-gp led to resistance to 17-DMAG in adenocarcinoma and large cell lung cancer cell lines. Paclitaxel-resistant cells were generated using A549 and H460 cell lines. These resistant cells acquired about 100-fold higher resistance to paclitaxel than the parental cells (Fig. 5a). Interestingly, induction of P-gp was observed in all paclitaxel-resistant cells, although H460/PR cells displayed a higher level of expression of P-gp than A549/PR cells (Fig. 5b). A cell survival assay showed that verapamil completely overcame paclitaxel resistance (Fig. 5c). These results demonstrated that the induction of P-gp plays a significant role in the acquisition of resistance to paclitaxel. As expected, paclitaxel-resistant cells showed cross-resistance to 17-DMAG, and the inhibition of P-gp restored sensitivity to 17-DMAG in paclitaxel-resistant cells (Fig. 5d-f). As shown in 17-DMAG-resistant cells, the induction of P-gp did not affect the sensitivity to AUY922 (Fig. 5g). These results further confirmed that P-gp expression is not associated with the efficacy of AUY922, but plays a major role in the mechanism of 17-DMAG resistance.

## Discussion

In our present study, we established three drug-resistant cell lines to investigate the mechanisms of acquired resistance to 17-DMAG in lung cancer with ALK rearrangement. We show from our findings that induction of P-gp expression is the main mechanism of resistance. In addition, we extend this finding to acquired resistance to paclitaxel.

The compound 17-DMAG is the first water-soluble analog of 17-AAG, has excellent bioavailability, and is quantitatively metabolized much less than is 17-AAG [28]. Thus, the mechanisms of acquired resistance to these drugs may be similar. NQO1 is a homodimeric metabolic enzyme that catalyzes the conversion of quinones to hydroquinones and has an important role in sensitivity to 17-AAG [29, 21]. Loss or low activity of NQO1, by the reduction of its mRNA or the emergence of inactivating polymorphisms in the *NQO1* gene, leads to resistance to 17-AAG in pancreatic cancer cells and glioblastoma cell lines [30, 21]. NQO1 expression is reduced in H3122/DR-1 and −2 cells, but not in H2228/DR cells. Nor do we find any differences in mRNA levels or DNA polymorphisms in *NQO1* between the parental and resistant cell lines. In addition, there are no significant changes in cell survival after treatment of the parental cells with dicumarol. These results suggest that NQO1 depletion is an unlikely resistance mechanism to 17-DMAG in cells with ALK rearrangement.

Hsp90 is a chaperone of client proteins relevant in NSCLC pathogenesis, including ALK and EGFR [31]. The inhibition of Hsp90 simultaneously disrupts these oncogenic signaling pathways, and consequently, cancer cell proliferation is inhibited by the induction of apoptosis or cell cycle arrest. Hsp90 inhibitors may be used to combat resistance to tyrosine kinase inhibitors (EGFR-TKIs and ALK inhibitors) regardless of secondary mutations [32, 12]. Hsp90 inhibitors inhibit the downstream effector pathways by controlling ALK through its degradation. We also observe that Hsp90 inhibitors sufficiently suppress the ALK signaling pathway in parental cells, but all 17-DMAG-resistant cells require higher concentrations of 17-DMAG to inhibit these pathways. Interestingly, resistant cells do not show cross-resistance to a different kind of Hsp90 inhibitor, AUY922, or to ALK tyrosine kinase inhibitors, crizotinib and TAE-684. These results imply that the resistant cells are still dependent on ALK signaling, and that acquisition of resistance to 17-DMAG may be caused by low intracellular 17-DMAG concentrations.

P-glycoprotein and multidrug resistance proteins (MRPs), ATP-binding cassette (ABC)-superfamily multidrug efflux pumps are responsible for some cases of chemoresistance. Expression of these pumps reduces cellular accumulation of cytostatic agents due to active efflux of these substrates [33–36]. The mRNA, protein, and activity of only one MRP family member *P-gp* is significantly induced in all 17-DMAG-resistant cells. Although verapamil pretreatment restores sensitivity to 17-DMAG in all resistant cells, a *P-gp*-specific siRNA was also used because verapamil can inhibit all MRP drug efflux pump proteins including P-gp. Similar to resistant cells, the inhibition of P-gp in the parental line H2228 enhances the sensitivity of cells to 17-DMAG, but not in the H3122 line. The baseline P-gp expression in the H2228 line may contribute to its slight resistance to 17-DMAG compared to H3122 cells. Therefore, we suggest that the induction of P-gp is associated with the primary or acquired resistance to 17-DMAG in cells with ALK rearrangement.

Induction of P-gp also leads to 17-DMAG resistance in other resistant cells. A number of drugs, such as taxol, doxorubicin, vincristine, VP-16, and cis-diamminedichloroplatinum (II), increase P-gp expression in lung cancer cell lines and animal models after chronic exposure [37–40]. Consistent with previous studies, we also detected induction of P-gp in cells with acquired resistance to paclitaxel. These resistant cells show cross-resistance to 17-DMAG, whilst the inhibition of P-gp restores the sensitivity to paclitaxel and 17-DMAG. Clinical evaluation of Hsp90 inhibitors, as single agents and in combination with various chemotherapy-agents, is currently in progress. Our findings suggest that P-gp expression should be considered in preclinical and clinical evaluation.

Overexpression of P-gp that recognizes a wide variety of chemotherapeutic agents and pumps them out of the cell is one of the principal causes of treatment failure in cancer. Diverse attempts are being made to overcome resistance via P-gp overexpression, although significant side effects remain a concern [41]. The four parental cell lines including H3122, H2228, A549, and H460 and cell lines resistant to 17-DMAG or paclitaxel showed persistent sensitivity to AUY922, a novel non-geldanamycin Hsp90 inhibitor. Consistent with our current results, previous studies have shown that AUY922 has effectiveness independent of P-gp expression [42, 21]. Thus, the treatment with new Hsp90 inhibitors may help overcome the acquired resistance to 17-DMAG caused by P-gp expression. A second alternative way to overcome resistance is through combination therapy; many drugs are known to inhibit the activity of P-gp [43–45]. We find that combined treatment with 17-DMAG and rapamycin overcomes drug resistance in 17-DMAG-resistant cells (Additional file 1). Previous studies have demonstrated rapamycin as a P-gp inhibitor [46, 47], and rapamycin is already approved for clinical use. Other types of Hsp90 inhibitors or a combination with additional therapeutic drugs, such as new P-gp inhibitors, are candidate strategies to overcome 17-DMAG-resistance caused by P-gp expression.

## Conclusions

In summary, the induction of P-gp expression may contribute to the acquired resistance to 17-DMAG in lung cancer cells with an ALK rearrangement. This resistance may be overcome by using a new Hsp90 inhibitor that is independent of P-gp expression or through a combined treatment with 17-DMAG and P-gp inhibitors.

## Additional file

Additional file 1: **Effects of treatment with a combination of 17-DMAG and rapamycin on parental and 17-DMAG-resistant cells.** Cells were treated with the indicated concentrations of 17-DMAG, rapamycin, or combination of two drugs for 72 h. Cell viability was measured 72 h later using the MTT assay. (TIFF 185 kb)

## Abbreviations

ALK: Anaplastic lymphoma kinase
17-DMAG: 17-(Dimethylaminoethylamino)-17-demethoxygeldanamycin
NQO1: NAD(P)H/quinone oxidoreductase 1
P-gp: P-glycoprotein
NSCLC: Non-small cell lung cancer
EML4-ALK: Echinoderm microtubule-associated protein-like 4 - anaplastic lymphoma kinase
EGFR: Epidermal growth factor receptor
c-MET: Mesenchymal epithelial transition growth factor
TKI: Tyrosine kinase inhibitor
HSP90: Heat shock protein 90
HSF1: Heat shock factor 1
17-AAG: 17-*N*-allylamino-17-demethoxygeldanamycin
MRPs: Multidrug resistance proteins

## References

[1] Soda M, Choi YL, Enomoto M, Takada S, Yamashita Y, Ishikawa S, Bando M, Ohno S, Ishikawa Y, Aburatani H, Niki T, Sohara Y, Sugiyama Y, Mano H (2007). Identification of the transforming EML4-ALK fusion gene in non-small-cell lung cancer. Nature.

[2] Shaw AT, Yeap BY, Mino-Kenudson M, Digumarthy SR, Costa DB, Heist RS, Solomon B, Stubbs H, Admane S, McDermott U, Settleman J, Kobayashi S, Mark EJ, Rodig SJ, Chirieac LR, Kwak EL, Lynch TJ, Iafrate AJ (2009). Clinical features and outcome of patients with non-small-cell lung cancer who harbor EML4-ALK. J Clin Oncol.

[3] Ettinger DS, Akerley W, Borghaei H, Chang AC, Cheney RT, Chirieac LR, D'Amico TA, Demmy TL, Govindan R, Grannis FW, Grant SC, Horn L, Jahan TM, Komaki R, Kong FM, Kris MG, Krug LM, Lackner RP, Lennes IT, Loo BW, Martins R, Otterson GA, Patel JD, Pinder-Schenck MC, Pisters KM, Reckamp K, Riely GJ, Rohren E, Shapiro TA, Swanson SJ, Tauer K, Wood DE, Yang SC, Gregory K, Hughes M (2013). Non-small cell lung cancer, version 2.2013. J Natl Compr Canc Netw.

[4] Shaw AT, Kim DW, Nakagawa K, Seto T, Crino L, Ahn MJ, De Pas T, Besse B, Solomon BJ, Blackhall F, Wu YL, Thomas M, O'Byrne KJ, Moro-Sibilot D, Camidge DR, Mok T, Hirsh V, Riely GJ, Iyer S, Tassell V, Polli A, Wilner KD, Janne PA (2013). Crizotinib versus chemotherapy in advanced ALK-positive lung cancer. N Engl J Med.

[5] Sasaki T, Koivunen J, Ogino A, Yanagita M, Nikiforow S, Zheng W, Lathan C, Marcoux JP, Du J, Okuda K, Capelletti M, Shimamura T, Ercan D, Stumpfova M, Xiao Y, Weremowicz S, Butaney M, Heon S, Wilner K, Christensen JG, Eck MJ, Wong KK, Lindeman N, Gray NS, Rodig SJ, Janne PA (2011). A novel ALK secondary mutation and EGFR signaling cause resistance to ALK kinase inhibitors. Cancer Res.

[6] Choi YL, Soda M, Yamashita Y, Ueno T, Takashima J, Nakajima T, Yatabe Y, Takeuchi K, Hamada T, Haruta H, Ishikawa Y, Kimura H, Mitsudomi T, Tanio Y, Mano H (2010). EML4-ALK mutations in lung cancer that confer resistance to ALK inhibitors. N Engl J Med.

[7] Katayama R, Shaw AT, Khan TM, Mino-Kenudson M, Solomon BJ, Halmos B, Jessop NA, Wain JC, Yeo AT, Benes C, Drew L, Saeh JC, Crosby K, Sequist LV, Iafrate AJ, Engelman JA (2012). Mechanisms of acquired crizotinib resistance in ALK-rearranged lung Cancers. Sci Transl Med.

[8] Kim HR, Kim WS, Choi YJ, Choi CM, Rho JK, Lee JC (2013). Epithelial-mesenchymal transition leads to crizotinib resistance in H2228 lung cancer cells with EML4-ALK translocation. Mol Oncol.

[9] Shaw AT, Kim DW, Mehra R, Tan DS, Felip E, Chow LQ, Camidge DR, Vansteenkiste J, Sharma S, De Pas T, Riely GJ, Solomon BJ, Wolf J, Thomas M, Schuler M, Liu G, Santoro A, Lau YY, Goldwasser M, Boral AL, Engelman JA (2014). Ceritinib in ALK-rearranged non-small-cell lung cancer. N Engl J Med.

[10] Solomon B, Wilner KD, Shaw AT (2014). Current status of targeted therapy for anaplastic lymphoma kinase-rearranged non-small cell lung cancer. Clin Pharmacol Ther.

[11] Katayama R, Khan TM, Benes C, Lifshits E, Ebi H, Rivera VM, Shakespeare WC, Iafrate AJ, Engelman JA, Shaw AT (2011). Therapeutic strategies to overcome crizotinib resistance in non-small cell lung cancers harboring the fusion oncogene EML4-ALK. Proc Natl Acad Sci U S A.

[12] Chen Z, Sasaki T, Tan X, Carretero J, Shimamura T, Li D, Xu C, Wang Y, Adelmant GO, Capelletti M, Lee HJ, Rodig SJ, Borgman C, Park SI, Kim HR, Padera R, Marto JA, Gray NS, Kung AL, Shapiro GI, Janne PA, Wong KK (2010). Inhibition of ALK, PI3K/MEK, and HSP90 in murine lung adenocarcinoma induced by EML4-ALK fusion oncogene. Cancer Res.

[13] Sequist LV, Gettinger S, Senzer NN, Martins RG, Janne PA, Lilenbaum R, Gray JE, Iafrate AJ, Katayama R, Hafeez N, Sweeney J, Walker JR, Fritz C, Ross RW, Grayzel D, Engelman JA, Borger DR, Paez G, Natale R (2010). Activity of IPI-504, a novel heat-shock protein 90 inhibitor, in patients with molecularly defined non-small-cell lung cancer. J Clin Oncol.

[14] Sang J, Acquaviva J, Friedland JC, Smith DL, Sequeira M, Zhang C, Jiang Q, Xue L, Lovly CM, Jimenez JP, Shaw AT, Doebele RC, He S, Bates RC, Camidge DR, Morris SW, El-Hariry I, Proia DA (2013). Targeted inhibition of the molecular chaperone Hsp90 overcomes ALK inhibitor resistance in non-small cell lung cancer. Cancer Discov.

[15] Normant E, Paez G, West KA, Lim AR, Slocum KL, Tunkey C, McDougall J, Wylie AA, Robison K, Caliri K, Palombella VJ, Fritz CC (2011). The Hsp90 inhibitor IPI-504 rapidly lowers EML4-ALK levels and induces tumor regression in ALK-driven NSCLC models. Oncogene.

[16] Socinski MA, Goldman J, El-Hariry I, Koczywas M, Vukovic V, Horn L, Paschold E, Salgia R, West H, Sequist LV, Bonomi P, Brahmer J, Chen LC, Sandler A, Belani CP, Webb T, Harper H, Huberman M, Ramalingam S, Wong KK, Teofilovici F, Guo W, Shapiro GI (2013). A multicenter phase II study of ganetespib monotherapy in patients with genotypically defined advanced non-small cell lung cancer. Clin Cancer Res.

[17] Carmichael J, DeGraff WG, Gazdar AF, Minna JD, Mitchell JB (1987). Evaluation of a tetrazolium-based semiautomated colorimetric assay: assessment of radiosensitivity. Cancer Res.

[18] Sameer AS, Shah ZA, Syeed N, Rasool R, Afroze D, Siddiqi MA (2010). NAD(P)H:quinone oxidoreductase 1 (NQO1) Pro187Ser polymorphism and colorectal cancer predisposition in the ethnic Kashmiri population. Asian Pac J Cancer Pre.

[19] Powers MV, Clarke PA, Workman P (2008). Dual targeting of HSC70 and HSP72 inhibits HSP90 function and induces tumor-specific apoptosis. Cancer Cell.

[20] McCollum AK, Teneyck CJ, Sauer BM, Toft DO, Erlichman C (2006). Up-regulation of heat shock protein 27 induces resistance to 17-allylamino-demethoxygeldanamycin through a glutathione-mediated mechanism. Cancer Res.

[21] Gaspar N, Sharp SY, Pacey S, Jones C, Walton M, Vassal G, Eccles S, Pearson A, Workman P (2009). Acquired resistance to 17-allylamino-17-demethoxygeldanamycin (17-AAG, tanespimycin) in glioblastoma cells. Cancer Res.

[22] Erlichman C (2009). Tanespimycin: the opportunities and challenges of targeting heat shock protein 90. Expert Opin Investig Drugs.

[23] Zhang H, Neely L, Lundgren K, Yang YC, Lough R, Timple N, Burrows F (2010). BIIB021, a synthetic Hsp90 inhibitor, has broad application against tumors with acquired multidrug resistance. Int J Cancer.

[24] Taldone T, Gozman A, Maharaj R, Chiosis G (2008). Targeting Hsp90: small-molecule inhibitors and their clinical development. Curr Opin Pharmacol.

[25] Benchekroun MN, Schneider E, Safa AR, Townsend AJ, Sinha BK (1994). Mechanisms of resistance to ansamycin antibiotics in human breast cancer cell lines. Mol Pharmacol.

[26] Romermann K, Wanek T, Bankstahl M, Bankstahl JP, Fedrowitz M, Muller M, Loscher W, Kuntner C, Langer O (2013). (R)-[(11)C]verapamil is selectively transported by murine and human P-glycoprotein at the blood–brain barrier, and not by MRP1 and BCRP. Nucl Med Biol.

[27] Achira M, Suzuki H, Ito K, Sugiyama Y (1999). Comparative studies to determine the selective inhibitors for P-glycoprotein and cytochrome P4503A4. AAPS pharmSci.

[28] Egorin MJ, Lagattuta TF, Hamburger DR, Covey JM, White KD, Musser SM, Eiseman JL (2002). Pharmacokinetics, tissue distribution, and metabolism of 17-(dimethylaminoethylamino)-17-demethoxygeldanamycin (NSC 707545) in CD2F1 mice and Fischer 344 rats. Cancer Chemother Pharmacol.

[29] Hadley KE, Hendricks DT (2014). Use of NQO1 status as a selective biomarker for oesophageal squamous cell carcinomas with greater sensitivity to 17-AAG. BMC Cancer.

[30] Siegel D, Shieh B, Yan C, Kepa JK, Ross D (2011). Role for NAD(P)H:quinone oxidoreductase 1 and manganese-dependent superoxide dismutase in 17-(allylamino)-17-demethoxygeldanamycin-induced heat shock protein 90 inhibition in pancreatic cancer cells. J Pharmacol Exp Ther.

[31] Shimamura T, Shapiro GI (2008). Heat shock protein 90 inhibition in lung cancer. J Thorac Oncol.

[32] Sawai A, Chandarlapaty S, Greulich H, Gonen M, Ye Q, Arteaga CL, Sellers W, Rosen N, Solit DB (2008). Inhibition of Hsp90 down-regulates mutant epidermal growth factor receptor (EGFR) expression and sensitizes EGFR mutant tumors to paclitaxel. Cancer Res.

[33] Taniguchi K, Wada M, Kohno K, Nakamura T, Kawabe T, Kawakami M, Kagotani K, Okumura K, Akiyama S, Kuwano M (1996). A human canalicular multispecific organic anion transporter (cMOAT) gene is overexpressed in cisplatin-resistant human cancer cell lines with decreased drug accumulation. Cancer Res.

[34] Schinkel AH, Mayer U, Wagenaar E, Mol CA, van Deemter L, Smit JJ, van der Valk MA, Voordouw AC, Spits H, van Tellingen O, Zijlmans JM, Fibbe WE, Borst P (1997). Normal viability and altered pharmacokinetics in mice lacking mdr1-type (drug-transporting) P-glycoproteins. Proc Natl Acad Sci U S A.

[35] Loe DW, Deeley RG, Cole SP (1998). Characterization of vincristine transport by the M(r) 190,000 multidrug resistance protein (MRP): evidence for cotransport with reduced glutathione. Cancer Res.

[36] Grant CE, Valdimarsson G, Hipfner DR, Almquist KC, Cole SP, Deeley RG (1994). Overexpression of multidrug resistance-associated protein (MRP) increases resistance to natural product drugs. Cancer Res.

[37] Young LC, Campling BG, Cole SP, Deeley RG, Gerlach JH (2001). Multidrug resistance proteins MRP3, MRP1, and MRP2 in lung cancer: correlation of protein levels with drug response and messenger RNA levels. Clin Cancer Res.

[38] Wang M, Hong X, Sun Q, Li R, Yang Z, Chen G (2012). [Establishment of animal model of a human lung adenocarcinoma drug-resistantcell line Anip973/NVB and investigation on mechanism of drug resistance]. Zhongguo Fei Ai Za Zhi.

[39] Melguizo C, Prados J, Luque R, Ortiz R, Caba O, Alvarez PJ, Gonzalez B, Aranega A (2012). Modulation of MDR1 and MRP3 gene expression in lung cancer cells after paclitaxel and carboplatin exposure. Int J Mol Sci.

[40] Horwitz SB, Cohen D, Rao S, Ringel I, Shen HJ, Yang CP (1993). Taxol: mechanisms of action and resistance. J Natl Cancer Inst Monogr.

[41] Binkhathlan Z, Lavasanifar A (2013). P-glycoprotein inhibition as a therapeutic approach for overcoming multidrug resistance in cancer: current status and future perspectives. Curr Cancer Drug Targets.

[42] Sharp SY, Prodromou C, Boxall K, Powers MV, Holmes JL, Box G, Matthews TP, Cheung KM, Kalusa A, James K, Hayes A, Hardcastle A, Dymock B, Brough PA, Barril X, Cansfield JE, Wright L, Surgenor A, Foloppe N, Hubbard RE, Aherne W, Pearl L, Jones K, McDonald E, Raynaud F, Eccles S, Drysdale M, Workman P (2007). Inhibition of the heat shock protein 90 molecular chaperone in vitro and in vivo by novel, synthetic, potent resorcinylic pyrazole/isoxazole amide analogues. Mol Cancer Ther.

[43] Zhang Y, Hsieh Y, Izumi T, Lin ET, Benet LZ (1998). Effects of ketoconazole on the intestinal metabolism, transport and oral bioavailability of K02, a novel vinylsulfone peptidomimetic cysteine protease inhibitor and a P450 3A, P-glycoprotein dual substrate, in male Sprague–Dawley rats. J Pharmacol Exp Ther.

[44] Sadeque AJ, Wandel C, He H, Shah S, Wood AJ (2000). Increased drug delivery to the brain by P-glycoprotein inhibition. Clin Pharmacol Ther.

[45] Qadir M, O'Loughlin KL, Fricke SM, Williamson NA, Greco WR, Minderman H, Baer MR (2005). Cyclosporin A is a broad-spectrum multidrug resistance modulator. Clin Cancer Res.

[46] Miller DS, Fricker G, Drewe J (1997). p-Glycoprotein-mediated transport of a fluorescent rapamycin derivative in renal proximal tubule. J Pharmacol Exp Ther.

[47] Lang R, Wagner H, Heeg K (1995). Differential effects of the immunosuppressive agents cyclosporine and leflunomide in vivo. Leflunomide blocks clonal T cell expansion yet allows production of lymphokines and manifestation of T cell-mediated shock. Transplantation.

