# Supplementary figures and images for: P-glycoprotein confers acquired resistance to 17-DMAG in lung cancers with an ALK rearrangement
Source: BMC Cancer. 2015 Jul 29;15:553. doi: 10.1186/s12885-015-1543-z (PMC4517346; doi:10.1186/s12885-015-1543-z)

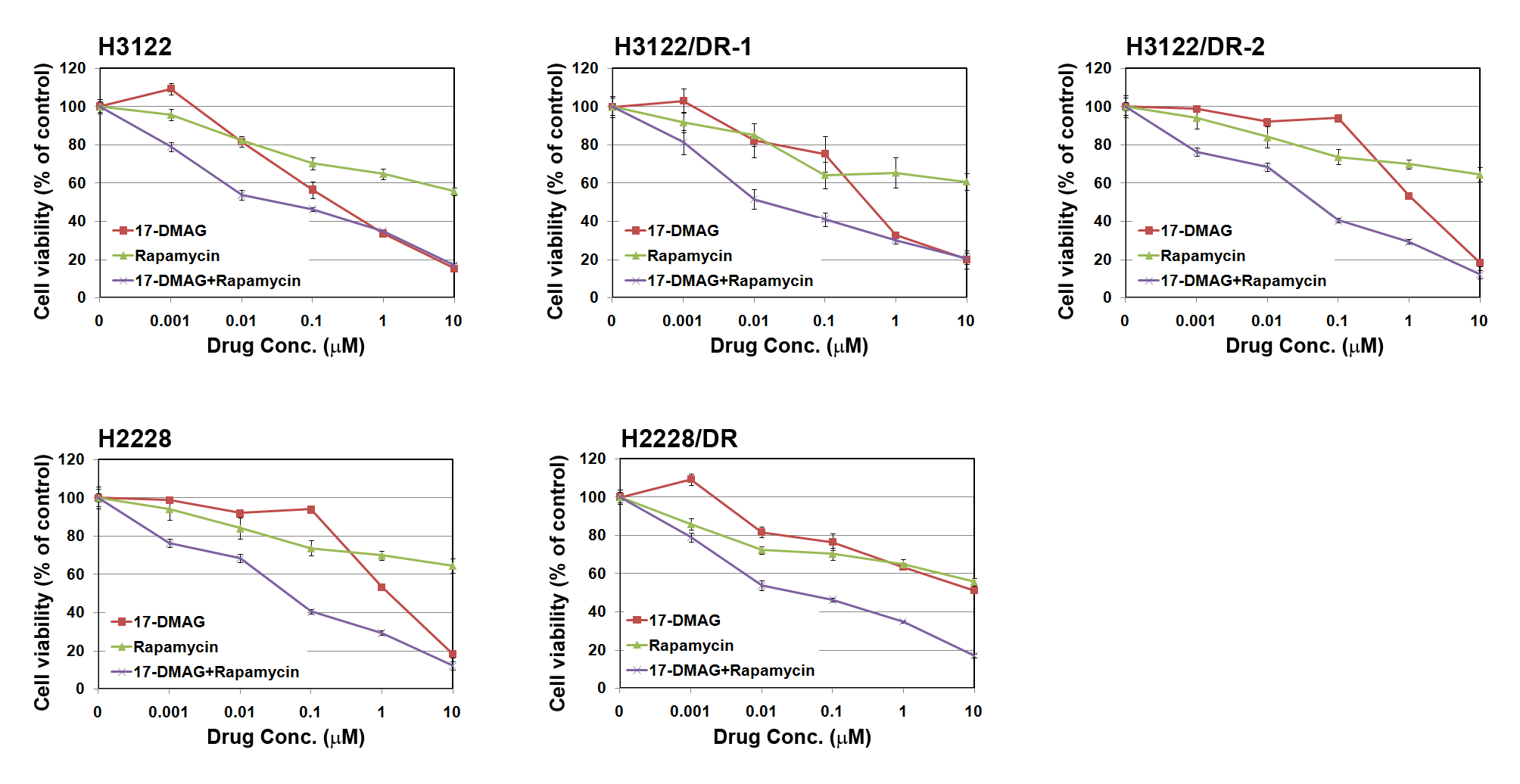

Supplement: Additional file 1: — Effects of treatment with a combination of 17-DMAG and rapamycin on parental and 17-DMAG-resistant cells. Cells were treated with the indicated concentrations of 17-DMAG, rapamycin, or combination of two drugs for 72 h. Cell viability was measured 72 h later using the MTT assay. (TIFF 185 kb) [file 12885_2015_1543_MOESM1_ESM.tiff]
